# Changing Stress Mindset Through Stressjam: A Virtual Reality Game Using Biofeedback

**DOI:** 10.1089/g4h.2018.0145

**Published:** 2019-09-18

**Authors:** Bernard M. Maarsingh, Jannah Bos, Charlotte F.J. Van Tuijn, Selwyn B. Renard

**Affiliations:** ^1^ University of Groningen, Department of Postmaster Education PPO, Groningen, The Netherlands.; ^2^ Jamzone, Leeuwarden, The Netherlands.; ^3^ Radboud University, Faculty of Social Sciences, Nijmegen, The Netherlands.; ^4^ Academic Medical Center University of Amsterdam, Amsterdam, The Netherlands.

**Keywords:** Heart rate variability, Virtual reality, Stress reduction, Game therapy

## Abstract

***Objective:*** A range of recent studies suggest that overall mindset about stress is related to health, performance, and well-being. Therefore, an exploratory study was conducted to examine whether virtual reality (VR) with real-time biofeedback would have potential in training people in an engaging way to develop a new stress-is-enhancing mindset.

***Materials and Methods:*** The specific application to improve people's stress mindset that was used in this study is Stressjam. The application was tested on its attractiveness by 111 healthy participants, specifically on their personal involvement through the Personal Involvement Inventory and its usability through the System Usability Scale. In addition to the healthy participants, a group of 64 patients dealing with stress used Stressjam for at least three sessions. The Stress Mindset Measure was used to assess the stress mindset of both groups, at baseline and after finishing their session(s).

***Results:*** Stressjam appears to be an application that is user friendly with good user involvement. The healthy participants and the patient sample both had a more positive stress mindset after using the application than at baseline, *t*(111) = 4.38, *P* < 0.001, and *F*(1,63) = 66.57, *P* < 0.001, respectively.

***Conclusion:*** The results of this study give some indications that using VR with biofeedback might be useful in working toward a more positive stress mindset. As such, further research into applications such as Stressjam is warranted.

## Introduction

Stress is generally perceived as a negative experience, and several studies show that stress can have negative effects on the brain and can cause structural changes to it, with effects on memory, cognition, and the way in which the stress response system works.^1,2^ Furthermore, increases in stress can inhibit the immune system, change the heartbeat, and activate the sympathetic nervous system, causing vasoconstriction, affecting blood pressure, blood lipids, and blood clotting, and even causing changes in vascular fibers.^3–5^ These changes can lead to cardiac rhythm disorders and, eventually, heart attacks.^4,6,7^ Stress can also affect the intestines, for example, Crohn's disease and irritable bowel syndrome are both associated with stress,^8,9^ and stress can cause various abnormalities in the function of the hormonal system.^10^

Aside from all the negative effects, stress also has a number of positive effects and the amygdala appears to play an important role in the way stress emerges.^11^ This seems to indicate that emotions, or more specifically the way in which people deal with stress emotionally is important. In recent years, this line of thought has gained more attention by researchers. For example, a series of studies suggests that a person's overall stress mindset plays a bigger role in health than stress itself.^12–16^ Stress mindset is defined as the overarching belief that stress is either enhancing or debilitating and as such is a distinct variable from traditional stress-related variables such as the amount, appraisal, and coping with stress.^13^ The importance of stress mindset is further shown by Keller et al. who found that individuals who believed stress negatively affects health were 43% more likely to die prematurely.^17^

Intriguingly, people with meaningful lives seem to worry more and have more stress than people with less meaningful lives. For those who live healthy lives despite high stress levels, stress appears to be an indicator of how engaged they are in activities that are personally meaningful rather than a sign of something being wrong.^18^ In line with this, research has shown the importance of specifically investigating the concept of stress mastery, which is the trainable capacity to learn to deal better with stress.^12–16^ For example, Crum and Lyddy showed that stress mindset can be changed through short film clips with information biased toward either the enhancing or the debilitating nature of stress.^13^ In addition, Kim et al. suggest that given that optimism is negatively related with numerous causes of mortality, targeting new optimism strategies to improve health is an important area for future research.^19^ It thus seems to be important to make someone's stress mindset more positive and personally meaningful.

As environmental variables and immediate contextual cues can influence behavior, changes in the environment may be an effective drive of behavioral change.^20^ A practical example of such an effect is that larger plates cause people to put between 28% and 32% more food on their plates.^21^

Furthermore, Thaler and Sunstein describe how desired healthy behavior might be trained by proactively designing the environment and using nudging strategies.^22^ However, how to use these factors to influence one's stress mindset has not been studied yet.

This study is aimed at exploring the attractiveness and effectiveness of changing one's stress mindset using virtual reality (VR), focusing on stress mastery, environmental changes, and nudging strategies. The use of VR allows for designing a world where stress and dealing with stress are central, and where stress-coping skills can be trained in a safe environment. The application Stressjam was tested, which is a VR game using biofeedback to measure stress that was specifically designed for this purpose. The attractiveness of Stressjam was assessed based on its user friendliness and through the user's personal involvement.

It was hypothesized that Stressjam can effectuate changes toward a stress-is-enhancing mindset by letting people experience that they need their stress to perform and advance in the game. Thus, the participant learns that stress can be helpful and sometimes necessary. In this study, we wanted to explore whether this VR application has the potential to influence stress mindset. In addition, it was hypothesized that Stressjam would be rated as user friendly with personal involvement.

## Materials and Methods

### Participants

Two different groups of participants were approached for this study. The first group consisted of healthy participants with no known physical or mental health problems (*n* = 111). These participants were recruited as a convenience sample through companies that had shown interest in Stressjam. This group participated in a single 1-hour session, and this part of the study was aimed at assessing whether Stressjam was an attractive game for users. The second group of participants consisted of a convenience sample of patients (*n* = 64) of a mental health facility that was already working with Stressjam. The patients were not included or excluded based on their diagnosis but could register themselves, in consultation with their therapist, as long as stress was considered a relevant issue in their treatment. These patients attended at least three sessions and this part of the study focused on exploring preliminary indications of Stressjams effectiveness.

### Instruments

Stressjam is an applied VR game with biofeedback, specifically heart rate variability (HRV). The HTC Vive was used for this study as VR hardware. In Stressjam, participants have an interactive VR experience on a tropical island allowing for several hours of gameplay. Stressjam has different levels and participants only get a few lives to achieve their in-game goals. The experience is personalized by using a HRV sensor on the chest. The game and biofeedback sensors are connected to communicate in real time to make the participants own stress system or, rather, their capacity to cope with stress, what is called in game terms their superpower. To advance in the game, the participant has to explore, find, and apply effective mechanisms in his/her own body to generate stress or remain calm, for example, only being able to open a gate by specifically becoming more or less stressed, for further details see Table 1.

**Table 1.** Characteristics of Stressjam

Health topic	Stress
Targeted age group	18–65 years
Other targeted group characteristics	Usable for clinical and nonclinical populations
Short description of game idea	The player starts in a building within a jungle environment. The in-game instructions guide the player through the game world while challenging the player to overcome obstacles such as needing to climb a rope to proceed using their stress system. The obstacles become harder as the player progresses in the game.
Target player	Individual
Guiding knowledge for behavior change	Nudging, HRV feedback training, rethinking stress mindset toolkit
Intended health behavior changes	Change the players stress mindset
Knowledge element to be learned	Stress can be helpful to overcome obstacles
Behavior change procedure employed	Conditioning, goal setting theory, health action process approach
Clinical or parental support needed?	No support needed as long as the player is capable of using VR
Data shared with parent or clinician	None is essential but summaries of HRV during the game and game performance can be shared
Type of game	Active, adventure
Story	
Synopsis	Players are on an exotic island, where they have to overcome various challenges to save the island from the problems caused by a volcano. The player should overcome the toxic elements of the island by using their stress system. Some challenges can be overcome by increasing their stress system, others by calming down.
How the story relates to targeted behavior change	Manipulating one's own stress level, measured through real-time HRV, is the only way the player can make progress and achieve the in-game goal.
Game components	
Player's game goal	Progress in the game using one's stress system
Rules	Everything is allowed within the game parameters
Game mechanic	
Procedures to generalize or transfer what is learned in the game to outside the game	Feedback canvas, based on deliberate practice in which daily life is translated to game goals and game results are translated in daily life goals.
Virtual environment	A jungle island with temples and other buildings
Setting	Fantasy
Avatar	
Characteristics	First person experience
Abilities	Move, pick things up, interact with objects
Game platform needed to play the game	Computer with VR equipment
Sensors used	HRV sensors
Estimated play time	3 hours

HRV, heart rate variability; VR, virtual reality.

HRV was used as a measure of stress as it is a good measure of stress itself but also because it appears to be an indicator of self-regulatory strength when it comes to stress.^23,24^ For this game, HRV is measured using the root mean square of successive differences (rMSSD), which gives a reliable and cost-efficient indication of the actual stress level.^25^ The game is designed in such a way that each participant plays using his or her own stress system. Their rMSSD scores are continuously kept track of >60 seconds, which produces the stress range displayed to the participant in-game. The average value for the previous 60 seconds is fed back to the participant and serves as a baseline. If the participant has a higher rMSSD value than that baseline for 6 seconds, they will see a blue color on their in-game controller (indicating calming down), and when they have a lower-than-average rMSSD, an orange color (indicating getting stressed) will appear. The more their stress level deviates from their average, the greater the result on the controller.

The Stress Mindset Measure (SMM-G) is an 8-item test to assess the participant's stress mindset. The SMM-G is scored on a 5-point Likert scale, ranging from 0, strongly disagree, to 4, strongly agree. A higher score indicates the participant experiences stress as a functional tool, a low score indicates the participant experiences stress as debilitating. The instrument has shown to be a reliable and valid instrument to test stress mindset.^13^

The System Usability Scale (SUS) is a 10-item questionnaire that uses a 5-point Likert scale ranging from strongly agree to strongly disagree to assess the user friendliness of an instrument. The SUS focuses on usability and learnability, with items such as “I thought the system was easy to use.” A score <51.0 is considered poor, a score between 51.0 and 80.3 is considered sufficient but improvable, and a score >80.3 is considered high. For example, a high score indicates that participants are likely to actively talk about the tested instrument.^26^

The Personal Involvement Inventory (PII) was used to assess participants' involvement. The PPI is a 10-item questionnaire with high internal consistency and test–retest reliability.^27^ Participants indicate on a 7-point scale the degree to which the application appeals to them on parameters such as being fascinating, interesting, appealing, and valuable. For example, a score of 1 would indicate the application being boring and a score of 7 would indicate it being interesting. Any negatively formulated items were transformed resulting in an average score between 10 and 70, with a higher score indicating a stronger personal involvement. A total score of >50 is considered high as this indicates an average score of 5 or higher on the individual items.

### Procedure

All participants gave and signed informed consent at the start of the study. The healthy participant took the SMM-G as premeasurement and the SMM-G, SUS, and PII as postmeasurement. The healthy participants played an 1-hour Stressjam session and were accompanied by staff for technical support during the game. Stressjam can be played without technical support but they were available for the purpose of this study. After the postmeasurement test, participants had the opportunity to examine what this hour of playing with their own stress system had done for them together with the staff member during a debriefing.

The researchers were blind to the diagnosis patients were being treated for. After registration, the SMM-G was taken as premeasurement and given again after each 1-hour session. Patients were free to stop whenever they wanted. A staff member was again available to provide technical support. Patients were asked to play three sessions for this part of the study, but they could play as many sessions as they wanted. All patients had a pregame and a postgame score on the SMM-G. This study was approved by the local ethics committee (RTPO Leeuwarden; RTPO1054).

### Analysis

The first part of the study, focusing on the attractiveness of Stressjam, examined whether the instrument met the criteria set with the PII and SUS. This was done through one sample *t*-tests. The second part focused on the difference between SMM-G scores before and after using Stressjam.

Although this part focused on the patient data, the data for healthy participants were also examined as it was available. For healthy participants, a paired sample *t*-test was performed, and for the patient data, a repeated measures analysis was used.

## Results

Exploratory analysis of the data showed that 62% of the healthy participants were female, whereas 52% of the patient sample was female. See Table 1 for baseline statistics of the two samples. Of the people who signed up for participation, there were no dropouts for the healthy sample. The patient sample started with an initial group of 64 participants, 21 continued for one additional session, 9 continued for 2 additional sessions, and 34 participants continued for 3 or more additional sessions. These additional sessions were a service to the participants and were thus not part of the research design or analyses (Table 2).

**Table 2.** Descriptive Statistics for the Healthy and Patient Samples

	*Healthy sample (*n* = 111)*		*Patient sample (*n* = 64)*	
	*M*	*SD*	*M*	*SD*
Percentage female	62	—	52	—
Age	43.0	10.5	40.6	11.5
SUS	80.5	10.6	—	—
PII	56.3	7.62	—	—
SMM-G baseline	2.11	0.66	1.59	0.49
SMM-G last measure	2.31	0.68	2.14	0.58

PII, Personal Involvement Inventory; SD, standard deviation; SMM-G, Stress Mindset Measure; SUS, System Usability Scale.

The first part of the study examined the attractiveness of Stressjam as reported by healthy participants. Participants on average scored 56.3 on involvement as measured with the PII. As this is significantly more than the 50 points requirement, this indicates participants were involved with the application, *t*(107) = 8.6, *P* < 0.001, 95% CI (54.85–57.76).

Participants had an average score of 80.5 on the SUS, which is just above excellent although not significantly so. With 95% confidence, the population value lies between 78.6 and 82.7 that is between above average and excellent. This score indicates that healthy participants considered the game to be user friendly in terms of both usability and learnability and is an indication that participants will actively tell others about the game.

The second part of the study focused on preliminary indications of the effectiveness of Stressjam. To test whether the patient sample, who signed up for stress-related problems, indeed had a less functional stress mindset than the healthy participants, an independent-samples *t*-test was conducted between these groups. There was a significant difference in scores between the psychiatric and the healthy participants at baseline, *t*(169) = 6.02, *P* < 0.001, 95% CI: 0.35–0.69. The psychiatric patients on average scored lower on the SMM-G, M = 1.59, standard deviation [SD] = 0.49, at baseline than employees, M = 2.11, SD = 0.66, confirming that psychiatric patients had a less positive stress mindset than nonpsychiatric patients.

**FIG. 1.**
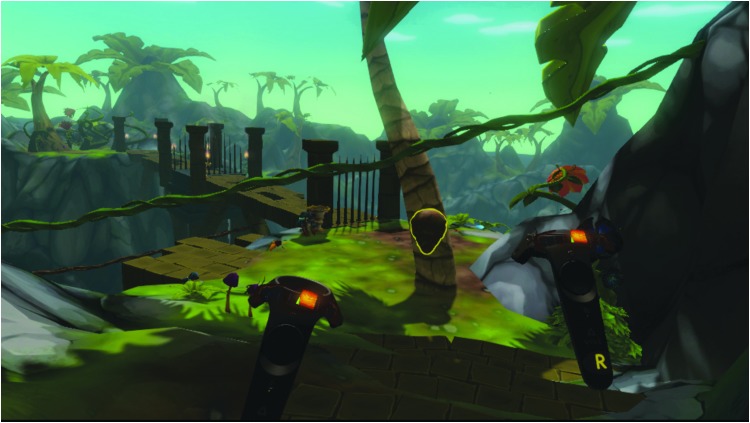
In-game world with the controllers indicating the participant is experiencing stress as measured through their heart rate variability.

A paired-samples *t*-test was conducted among healthy participants to compare the SMM-G at baseline and after playing Stressjam for I hour. The healthy participants showed a significantly more positive mindset toward stress after playing Stressjam for 1 hour, *t*(111) = 4.38, *P* < 0.001. For the patient sample, the repeated measures analysis showed a significant linear change over the course of the three Stressjam sessions, *F*(1,63) = 66.57, *P* < 0.001. Patients on average had a more positive stress mindset at the end of the three sessions than at the start.

## Discussion

The aim of this study was twofold, to examine whether using VR with biofeedback, specifically Stressjam, is related with improvements in the stress mindset of participants, and to examine the attractiveness of the application to participants. To this end, two groups were tested, a healthy sample and a patient sample. The group of healthy participants was offered one session of Stressjam, and although stress mindset was examined, the focus of this part of the study was on degree of involvement and user friendliness. Participants assessed Stressjam as an application that is user friendly and that elicits personal involvement, both cognitively and emotionally. This makes Stressjam an attractive application for further research into its effectiveness in helping people incorporate a more positive stress mindset.

In the second part of the study, the focus was on exploring whether there were any indications that Stressjam might have a positive effect on one's stress mindset. It is important to emphasize that this study did not explore any causality as there was no control group. However, the results do show that both the healthy participants and the patient group showed a more positive stress mindset after participating in Stressjam than at the start of it. These findings are in line with previous research suggesting that stress mindset can be changed through intervention.^15^ If these effects can indeed be attributed to Stressjam, this would be the first study to indicate the possibility to help people adopt a more positive stress mindset by using a VR game with biofeedback.

There are several limitations that should be addressed. First and foremost the design of this study was exploratory in nature and as such only contained participants who were self-motivated to play Stressjam, this could mean that the obtained results were more positive than would have been found for a random sample. Second, some of the clinicians indicated being skeptical toward using VR, which could have influenced who ended up entering the study. The impact of this study is further limited by the fact that the study did not involve a control group, and as such it cannot be concluded that using Stressjam caused the mindset improvement that was found over time. Third, variables such as regression to the mean, a placebo effect, and artifacts cannot be ruled out. As there was no long-term follow-up, any changes that were found may have quickly disappeared. The results may also be biased as they are based on self-reports, participants may have been aware of the purpose of the study and answered the questionnaires accordingly.

The promising results of this study have already led to several other studies, for example, a randomized clinical trial will start in the Netherlands to test whether the results of this study can indeed be attributed to Stressjam. Furthermore, to get a better picture of how to train self-regulatory strength, a study will be conducted on multiple offshore drilling platforms inquiring into employees' health, with HRV playing an important role.

A second line of research focuses on various different populations that might benefit from a training focused on creating a more positive stress mindset. For example, a study will specifically examine patients with addiction problems and another study focuses more on preventing stress-related problems and, as Crum recommends, magnifying cognitive, emotional, and physical attributes that may contribute to better adaptive responses over time.^13^

Future research may also want to examine to what extent VR is needed or whether similar effects can be obtained without using VR. In this study, VR was used as it allows for a more lifelike experience as participants are more or less in the game, but there are also some downsides to VR such as some participants getting motion sick in VR and the added cost of the VR equipment. In addition to looking at any long-term effects and whether some form of continuous training is needed to retain the effects, it would be interesting to examine how many sessions would lead to the best results.

In conclusion, training people to adopt a stress-is-enhancing mindset is relevant, as research has shown that such a mindset results in major health benefits.^18^ This study suggests that Stressjam is an interesting application to further study with regard to its effectiveness. However, further research needs to be done before Stressjam in clinical practice can be recommend.
